# Development of a Novel Formulation That Improves Preclinical Bioavailability of Tenofovir Disoproxil Fumarate

**DOI:** 10.1016/j.xphs.2016.12.003

**Published:** 2017-03

**Authors:** Melynda E. Watkins, Steve Wring, Ryan Randolph, Seonghee Park, Kendall Powell, Lissa Lutz, Michelle Nowakowski, Ram Ramabhadran, Paul L. Domanico

**Affiliations:** 1 The Clinton Health Access Initiative, Research and Development, Boston, Massachusetts 02127; 2 SCYNEXIS, Inc., Jersey City, New Jersey 07302

**Keywords:** bioavailability, Caco-2 cells, intestinal absorption, intestinal metabolism, membrane transporter, prodrugs

## Abstract

Tenofovir disoproxil fumarate (TDF), the bisphosphonate ester prodrug of tenofovir (TFV), has poor bioavailability due to intestinal degradation and efflux transport. Reformulation using U.S. Food and Drug Administration–approved esterase and efflux inhibitors to increase oral bioavailability could provide lower dose alternatives and reduce costs for patients with HIV in resource-limited settings. Inhibition of mucosal and intracellular esterases was studied in human and rat intestinal extracts (S9), where TDF was protected by the carboxylesterase inhibitor bis-para-nitrophenylphosphate, the ester mix EM1, and the generally recognized-as-safe (GRAS) excipient propylparaben. Permeability studies using Madin-Darby canine kidney and Caco-2 cell monolayers demonstrated that TDF was a substrate for the permeability glycoprotein with permeability glycoprotein inhibitors reducing basolateral to apical transport of TDF. These studies also showed that transport was increased by esterase inhibitors. TDF, TFV, and tenofovir monophosphonate ester transport across Caco-2 monolayers with esterase and efflux inhibitors revealed a maximum 38.7-fold increase in apical to basolateral TDF transport with the potent non-GRAS combination of EM1 and GF120918. Transport was increased 22.8-fold by the GRAS excipients, propylparaben, and d-a-tocopheryl polyethylene glycol 1000 succinate (a vitamin E derivative). TFV pharmacokinetics in rats following oral administration of TDF and GRAS esterase and efflux inhibitors confirmed enhanced bioavailability. Area under the curve increased 1.5- to 2.1-fold with various combinations of parabens and d-a-tocopheryl polyethylene glycol 1000 succinate. This significant inhibition of TDF hydrolysis and efflux *in vivo* exhibits the potential to safely increase TDF bioavailability in humans.

## Introduction

Approximately 37 million people worldwide were living with HIV/AIDS in 2015, and as of June 2015, less than half of them had access to antiretroviral therapy (ART).^1^ Initiatives such as 90-90-90^2^ and Test and Treat^3^ have set the stage for 90% of patients with diagnosed HIV to be placed onto sustained ART by 2020. To avoid a financial crisis in the clinical management of 37 million people living with HIV/AIDS, the global health community is exploring every option possible to improve healthcare efficiencies.

The first Conference on Antiretroviral Dose Optimization (CADO)^4^ identified several realistic opportunities to refine treatment regimens through improvements in process chemistry, product dosage formulation, and dose optimization. One of CADO’s top priorities was to reformulate tenofovir disoproxil fumarate (TDF) to increase the product’s bioavailability.

TDF, the prodrug of tenofovir (TFV), a potent, well-tolerated nucleoside reverse-transcriptase inhibitor (NRTI), is usually prescribed for first-line ART as part of a fixed-dose combination once-daily treatment containing TDF (300 mg), lamivudine (300 mg), or emtricitabine (200 mg), and efavirenz (600 mg).^5^ In 2014, the total number of first-line patients on a TDF-containing regimen in resource-limited settings (RLS) was 8.3 million,^6^, ^7^ which at the time constituted more than half of all patients worldwide on treatment.

TFV has very low intestinal permeability due to its highly charged phosphonate group, thereby eliminating its use as an orally administered drug. In turn, TDF was designed to improve oral bioavailability. Oral administration of TDF was shown to increase bioavailability of TFV to 20% in mice,^8^ 30% in dogs,^9^ and slightly under 30% in humans.^10^, ^11^, ^12^

*In vitro* studies have demonstrated that TDF is hydrolyzed to TFV in 2 steps with the tenofovir monophosphonate ester (TFV-ME) as the intermediate metabolite. The first step is catalyzed by carboxylesterases (CE) and the second by phosphodiesterases (PDEs).^8^ TFV is converted to its active bisphosphorylated moiety by intracellular kinases.^13^ TFV’s sustained potency is due to its long intracellular half-life of 33-50 h in resting lymphocytes and 12-15 h in activated lymphocytes.^14^

Despite the advantages conferred by the prodrug TDF, the bioavailability of systemic TFV remains limited. The acceptable but modest bioavailability of TDF represents an opportunity through reformulation to reduce dose while maintaining efficacy and generating significant cost savings. Our group has extensive experience with costing commodity drugs for RLS. We can calculate with reasonable certainty how much of a product’s price is due to raw materials, active pharmaceutical ingredient manufacturing, formulation, overhead, and profit. Given the size of the market for products containing TDF, we determined that a 33% improvement in bioavailability resulting in a reduction in the standard 300 mg TDF dose to 225 mg would lead to $50-75 million in annual savings (CHAI Market Analysis, internal). Furthermore, demonstrating a positive outcome from a clinical proof of concept could guide similar research efforts for other products being used today in RLS.

TDF, as with all orally administered products, faces a variety of pharmacological obstacles before reaching systemic circulation. The product is first subject to the acidic environment of the stomach. It then passes into the duodenum where it is exposed to pancreatic and luminal enzymes as well as higher pHs in the intestinal milieu.^15^, ^16^ During absorption by the small intestine, the product is exposed to mucosal esterases and various efflux transporter systems that include P-glycoprotein (P-gp).^17^, ^18^ Finally, whatever TDF remains and reaches the serosa is readily hydrolyzed by cytosolic, plasma, and liver esterases.^8^ Each of these obstacles offers a potential opportunity to protect TDF and increase its bioavailability by ensuring stability across a wider pH range, minimizing enzymatic degradation, and blocking P-gp-mediated efflux. This study investigates such opportunities in order to identify a novel, safe, and cost-effective formulation that could substantially improve its bioavailability in humans.

Earlier pH studies indicate that ester prodrugs such as TDF are stable between pH 2 and pH 6, but become increasingly sensitive to chemical hydrolysis as pH rises above pH 6.^8^, ^19^ As noted, the intestine receives pancreatic secretions containing a mix of amylases, proteases, and lipases. An increase in estimated TDF bioavailability from 25% when fasting to 39% following a high fat meal^10^, ^11^, ^12^ suggests that competitive inhibition of lipases by food may provide a degree of protection. This investigation assessed the stability of TDF in simulated gastric and intestinal milieus, including the presence of pancreatic lipases, in order to identify competitive inhibitors capable of improving stability in these environments.

TDF is subject to CE and PDE-mediated enzymatic degradation at the luminal, mucosal, and intracellular levels.^8^ Rapid hydrolysis of the prodrug was observed previously in intestinal homogenates from rat, pig, and human, and Caco-2 cell extracts, and this degradation was inhibited by the addition of ester-containing fruit extracts.^20^ The addition of discrete esters and ester mixtures to rat intestinal homogenates also demonstrated a stabilizing effect on TDF; in particular, propylparaben (PP) and PP-containing mixtures showed almost complete (94.5% and 98.5%, respectively) inhibition of TDF metabolism.^21^ Coincubation of ester mixtures and TDF resulted in enhanced absorption across Caco-2 monolayers and in rat ileum.^21^ This increase in the amount of intact prodrug on the basolateral or mesenteric side indicates effective inhibition of TDF degradation by the added esters. Studies comparing various intestinal homogenates also demonstrated that rat ileum had esterase activity similar to human ileum,^22^ making it an appropriate model for studies on the intestinal degradation of TDF. This study sought to compare the effects of esterase and other enzyme inhibitors on TDF metabolism in human and rat intestinal homogenates.

Absorption and bioavailability of orally administered drugs is complicated by the role of efflux transporters, such as P-gp coded by *ABCB1*, that use their broad substrate specificity to return many drugs back into the intestinal lumen.^17^, ^18^ Known inhibitors of P-gp such as the acridone carboxamide derivative GF120918 (GF918)^23^ and cyclosporin A (CsA)^24^ can be used to measure efflux effects on specific compounds across monolayer systems such as MDR1-Madin-Darby canine kidney (MDCK) and Caco-2 cells. While initial reports suggested that TDF is unlikely to be affected by P-gp inhibitors,^25^ later studies have shown that P-gp or adenosine triphosphate–binding cassette transporters are involved in efflux transport of TDF across Caco-2 systems.^21^ Esterase inhibitors in conjunction with CsA enhanced transport of TDF across Caco-2 monolayers more than either inhibitor alone, suggesting additive or synergistic effects. Increased transport of TDF across both Caco-2 and MDCK monolayers was also observed in the presence of protease inhibitors that inhibited P-gp.^26^ This study assessed both the intrinsic permeability of TDF and its monoester form in MDCK systems as well as the relative contribution of P-gp efflux transport on permeability. This study then utilized the Caco-2 system to elucidate dose-dependency relationships between TDF efflux and known inhibitors of the transport protein.

The key objective of this investigation was to determine the feasibility of substantially improving the oral bioavailability of TDF by using approaches appropriate for eventual use in humans to treat people living with HIV/AIDS in RLS. Specifically, this body of work examined TDF metabolism and efflux transport to identify targets that might increase the fraction of TFV absorbed. These studies explored the stability in gastric and intestinal environments both with and without pancreatic enzymes, the hydrolysis by intraluminal and enterocytic esterases, and the restrictive absorptive permeability and efflux transport by P-gp across MDCK and Caco-2 monolayer systems. They also sought to establish the dynamic range for potential optimization of TDF absorption from using various pharmacological agents and to identify a combination of generally recognized-as-safe (GRAS) excipients that could improve the absorption of TDF to a significant degree by inhibiting both metabolism and efflux transport. Finally, the oral bioavailability of the optimized GRAS formulation was evaluated *in vivo* using rats as final confirmation prior to the launch of human clinical studies.

## Materials and Methods

### Materials

TDF was synthesized in the CHAI laboratory in West Chester, PA. TFV-ME and GF918 were synthesized by SCYNEXIS. TFV (Selleckchem) and TFV-d7 (TLC Pharmaceutical Standards Ltd.) were purchased. Bis-para-nitrophenylphosphate (BNPP), phenylmethylsulfonyl fluoride (PMSF), PP, methylparaben (MP), ethylparaben (EP), ethyl octanoate, octyl acetate, *p*-nitrophenyl acetate, propranolol, amprenavir, and d-a-tocopheryl polyethylene glycol 1000 succinate (TPGS) were purchased from Sigma-Aldrich, whereas butylparaben (BP) was purchased from Fisher Scientific. Transport medium consisted of Hanks’ balanced salt solution (HBSS) adjusted to a pH of 7.4. Potassium ethylenediaminetetraacetic acid (EDTA) rat plasma for calibration sample preparations was purchased from Bioreclamation (Westbury, NY). PMSF-free rat and human intestinal S9 fractions were purchased from Xenotech (Lenexa, KS). Porcine pancrelipase (PL) (lipase activity: 26.9 U/mg; protease activity: 146 U/mg; amylase activity: 158 U/mg) was purchased from MP Biomedicals, LLC. All solvents were HPLC or reagent grade and obtained from Fisher Scientific.

### pH Stability

The pH of fasted state simulated intestinal fluid (FaSSIF)^27^ was adjusted with 2M NaOH to pH of 6.8, 9, and 11. Simulated gastric fluid (SGF) was used as is at pH 1.2. TDF solutions (100 mM) were prepared in 99% dimethyl sulfoxide. TDF solutions (1 mM) were prepared by 100-fold dilution in FaSSIF and SGF. The pH of the final solution was not measured.

The TDF stability study at pH 1.2 was measured at 37°C in triplicate at 0, 15, 30, 60, 90, and 120 min. The TDF stability studies at pH 6.8, 9, and 11 were measured at 37°C in triplicate at 0, 30, 60, 120, and 240 min. Samples (5 μL) were quenched by diluting 200-fold with 4°C 0.1% formic acid (35% MeOH). These samples were analyzed by liquid chromatography tandem mass spectrometry (LC-MS/MS).

### Stability in SGF and FaSSIF With and Without PL

TDF and TFV-ME solutions were prepared in duplicate in either SGF or FaSSIF with and without porcine PL enzymes to give final concentrations for the TFV species of 10 μM and for PL of 480 U/mL. Samples were incubated at 37°C and quenched with acetonitrile at 0, 15, 30, 60, 90, and 120 min. For each time point, the % TDF or % TFV-ME remaining was determined by LC-MS/MS as described below.

### Hydrolysis of TDF by PL and Effect of Enzyme Inhibitors

TDF solutions were prepared at 10 μM and incubated in triplicate at 37°C in FaSSIF containing PL at 480 U/mL with and without inhibitors. Inhibitors were added at 10% vol/vol for the oils (sesame, olive, safflower, soybean, and coconut); at 10 mM for PP, MP, and EM1; and at 0.05 mM/0.002 mM for EDTA/PMSF. Controls consisted of TDF in transport media or FaSSIF with and without PL. Samples were incubated at 37°C and quenched with acetonitrile at 10, 15, 20, and 30 min. For each time point, the % TDF or % TFV-ME remaining was determined by LC-MS/MS as described below.

### Rat and Human Intestinal S9 Fractions

TDF solutions were prepared at 10 μM in rat or human intestinal S9 fraction (0.5 mg protein/mL). Inhibitors were added to the solutions. The inhibitors studied were BNPP (10 μM), PMSF (100 μM), EM1 (0.05% wt/vol), PP (1 mM), MP (1 mM), and EP (1 mM). Control samples included buffer, human S9, or rat S9 solutions with only TDF. Samples were incubated at 37°C for 0, 5, 10, 15, 20, and 30 min and then analyzed by LC-MS/MS for TDF, TFV-ME, TFV, and PP (to quantify stability).

### MDCK-MDR1 Efflux Studies

MDCK-MDR1 cells (Netherlands Cancer Institute) were seeded at a density of 3 × 10^5^ cells per well onto microporous polycarbonate membranes in 12-well Costar Transwell plates (Corning, Inc.). The cells were incubated for 3 days during which time they formed confluent monolayers. Monolayer integrity was checked using a Millicell-ERS instrument (Millipore) to determine the transepithelial electrical resistance (TEER) across the monolayer. The absorptive permeability and susceptibility for P-gp-mediated efflux was evaluated in separate experiments by adding TDF or TVF-ME at concentration of 5 μM in the presence or absence of 2 μM GF918. All test compounds were added to the apical compartment. Competency of the P-gp efflux transporter was confirmed by assay of propranolol (nonsubstrate) and amprenavir (substrate). Cell monolayers were incubated in triplicate with shaking (160 rpm) at 37°C for 2 h. Samples were removed from the apical and basolateral compartments after incubation and assayed for test compound concentrations by LC-MS/MS. Values for mass balance, *P*_app, A>B_, *P*_app, A>B_ + GF918, and absorption quotient were calculated for each compound.^28^, ^29^

### Caco-2: Determination of Permeation Through Caco-2 Cell Monolayers

Caco-2 monolayer experiments were performed as previously described^30^ with a few modifications. When confluent, Caco-2 cells were removed from cell culture flasks and seeded onto polycarbonate membrane transwells at a density of 5 × 10^5^ cells/cm^2^ (Dulbecco's Modified Eagle's medium, 15% vol/vol fetal calf serum) and incubated (37°C, 5% CO_2_) for 24 h. Following incubation, the media was replaced to remove dead cells and prevent the formation of multiple layers of cells settling on the filter. Media was then changed every 2-3 days and plates were used in experiments after a total of 21 days from initial seeding. Monolayer integrity was checked before and after each experiment using a Millicell-ERS instrument to determine the TEER across the monolayer and to ensure that there was no substantial change in monolayer integrity throughout the duration of an experiment. On the day of the experiment, the TEER was assessed and the media in each plate was replaced with warm HBSS (pH 7) and allowed to equilibrate (37°C, 30 min). Apical (for apical-to-basolateral [A-to-B] transport) chambers were replaced with HBSS containing 10 μM (final concentration, *n* = 3 reps) aqueous solution of drug and inhibitors. Samples were taken from both apical and basolateral compartments at 120 min and diluted into an appropriate solvent to allow drug quantitation using LC-MS/MS. Calculations were performed as described in the previous section.

### Rat Pharmacokinetics

Male Sprague-Dawley rats, each weighing approximately 350-400 g, were obtained by ViviSource. The animals were fasted overnight prior to dosing and for 2 h postdose. Water was offered *ad libitum* throughout the study. Animals (6 per group) were dosed with a slurry containing 25 mg/kg TDF and various inhibitors. Inhibitor concentrations were 40 mg/kg for BP, EP, MP, or PP; 100 mg/kg for TPGS; 8 mg/kg for EM1; and 3 mg/kg for GF918. During the dose ranging studies, PP was dosed at 40, 120, and 400 mg/kg. The dose vehicle consisted of 10% ethanol, 30% polyethyleneglycol-400, 30% propylene glycol, 0.05% carboxymethylcellulose, and 30% water. The formulation was administered at a dose rate of 2 mL/kg. Blood samples were taken at 20, 40, 60, 80, 100, 120 min and then at 4 and 8 h. Two hours after dosing, food was supplied per ViviSource standard operating procedures. The in-life phase of the study, including generation of plasma samples, was performed by ViviSource, while bioanalysis and calculation of pharmacokinetics (PK) parameters was performed by SCYNEXIS. Descriptive PK parameters (*T*_max_, *C*_max_, and area under the curve [AUC]) were calculated by means of noncompartmental analysis in Microsoft Excel. Values for AUC were determined using the linear trapezoidal method. The protocols were approved by the Institutional Animal Care and Use Committee prior to study start. The animals were cared for in accordance with the Animal Welfare Act, the Guide for the Care and Use of Laboratory Animals, and the U.S. Office of Laboratory Animal Welfare. Medical treatment necessary to prevent unacceptable pain and suffering, including euthanasia, was the sole responsibility of the attending laboratory animal veterinarian.

### Sample Preparation and LC-MS/MS Analysis for All Samples

The LC-MS/MS system consisted of an LEAP autosampler, an Agilent 1100 series liquid chromatograph, and a SCIEX 4000 QTRAP^®^ mass spectrometer (or equivalent) operated in triple quadrupole mode. A Phenomenex Synergi™ Polar-RP column (4 μm, 2.1 × 150 mm) at 60°C was used with 3/1/96 acetonitrile/acetic acid/water as the mobile phase. Isocratic elution was performed at a flow rate of 0.6 mL/min. The mass spectrometer was operated in positive Turbo IonSpray™ mode with multiple reaction monitoring. Q1/Q3 ions for TFV and TFV-d7 were 288.1/176.1 and 295.2/183.0, respectively. DP, CE, and CXP were typically 80, 35, and 11, respectively, for both analyte and internal standard. The source temperature was 550°C, and the voltage was 3500 V.

Following LC-MS/MS analysis, peak area ratios were calculated (analyte peak area divided by internal standard peak area). Standard curves were created by least squares fitting plots of peak area ratio versus nominal concentration. Sample concentrations were calculated from the results of least squares fits. For rat plasma PK study samples, accuracy acceptance criteria for standards and quality control samples were ±15% of nominal except for lower limit of quantification which was ±20%. For *in vitro* studies where calibrators were used, accuracy acceptance criteria were ±20% across the whole concentration range.

#### Rat Plasma Sample Bioanalysis

Stock solutions of TFV (Selleckchem) and TFV-d7 (TLC Pharmaceutical Standards, Ltd) were each prepared at a concentration of 1 mg/mL in water and stored at −20°C. Calibration curves were constructed by serial dilution of the TFV stock using EDTA rat plasma (Bioreclamation) as the diluent. The final calibrator concentrations were 4.92, 12.3, 30.7, 76.8, 192, 480, 1200, and 3000 ng/mL. Quality control samples were prepared in the same manner at the following concentrations: 15.0, 150, 2,400, and 10,000 ng/mL.

Samples (e.g., blanks, standards, quality control samples, and study samples) were extracted using a protein precipitation procedure. A 25 μL aliquot of rat plasma was aliquoted into a deep well 96-well plate. To the sample aliquot, 10.0 μL of an internal standard solution was added. The internal standard solution contained TFV-d7 at 500 ng/mL in water. Blanks used 10 μL of neat water in lieu of the internal standard solution. Neat methanol (150 μL) was also added to each well. The 96-well plate was capped, shaken for 10 min, and centrifuged at 4000 relative centrifugal force for 10 min at 15°C. A 120 μL aliquot of the resulting supernatant was transferred to a clean, deep well 96-well plate and evaporated to dryness (N_2_, 40°C) using a TurboVap96™ evaporator (Biotage). All samples were reconstituted with 100 μL mobile phase (see below), mixed, and transferred to a microliter 96-well plate for LC-MS/MS analysis.

#### Permeability Sample Analysis (Caco-2)

Stock solutions of TDF (CHAI) and TFV-ME (SCYNEXIS) were prepared at 10 mM in dimethyl sulfoxide and stored at −20°C. Independent calibration curves from 0.2 to 500 nM of TFV, TFV-ME, and TDF were prepared by serial dilution of stocks in Mobile Phase A (see below). Samples from permeability assays were also diluted into Mobile Phase A prior to analysis.

The LC-MS/MS system and conditions were as described above with the following exceptions. Mobile Phase A was 3/1/96 acetonitrile/acetic acid/water and Mobile Phase B was 96/1/3 acetonitrile/acetic acid/water. Rather than isocratic elution, a gradient program was used. The gradient program held at 0% B for 1 min, ramped to 98% B from 1 to 2 min, held at 98% B from 2 to 3 min, and then returned to initial conditions. Q1/Q3 ions for TFV-ME and TDF were 404.0/270.0 and 520.1/270.0, respectively.

#### Permeability Sample Analysis (MDCK)

Conditions were similar to those used for Caco-2 analyses. Differences included the use of a Phenomenex Synergi Hydro-RP column (4 μm, 2.1 × 50 mm) at 60°C, mobile phases of aqueous 0.2% formic acid (A) and neat methanol (B), and a rapid 0.9 min gradient from 0% to 95% B.

#### Other *In Vitro* Sample Analysis

Samples from S9 incubations were analyzed using the LC-MS/MS conditions described earlier for Caco-2 samples. Samples from pH stability studies were typically analyzed using a Thermo Scientific AQUASIL C18 Dash HTS column (5 μm, 2.1 × 20 mm), a mobile phase A of aqueous 5 mM ammonium formate with 0.1% formic acid, and a rapid 1.5 min gradient from 5% to 95% B. Mobile phase B was either methanolic 5 mM ammonium formate with 0.1% formic acid or neat acetonitrile.

### Data Analysis

Experimental replicates are represented in graphs as their mean and SD. Time series studies were fit using JMP (version 12, SAS Institute, Inc) to a two-parameter exponential decay [*y* = scale × exp (rate × time)]. Fit parameters were calculated using all replicates to reduce estimate variability. Results using this model showed little systematic error. The decay rate constants were transformed into half-lives and errors in half-lives were calculated using fractional error analysis from the estimate parameters and their standard errors.

$$Error\;in\;half\;life=\frac{S\tan dard\;Error_{decay\;rate}}{decay\;rate}*half\;life$$

Uncertainties are reported as the SEM and are also shown as 95% confidence intervals (95% CI). AUC values were normalized to both dose and animal weight. Box-and-whisker plots are included in the Caco-2 permeability and rat AUC studies to graphically show experimental variability. The ends of each box represent the first and third quartiles. The horizontal line within the box represents the median sample value. The whiskers extending from the ends of each box are computed as first quartile − 1.5 × (interquartile range) and third quartile + 1.5 × (interquartile range), where the interquartile range is the difference between the first and third quartiles.

Median values were used to rank pharmacological agents on their ability to affect TDF uptake in rats. We ranked agents by their median versus their mean since the median is more resilient to large or small values that may skew the mean, thus reducing the impact of outliers on analyses. As such, and in these circumstances, the median is a better descriptor of a “typical” outcome.

## Results

### Pharmacological Agent Strategy

Several groups of pharmacological agents were used in these studies. The first group included active pharmaceutical ingredients (GF918, CsA) and various compounds (BNPP, EM1, PMSF) that are excellent pharmacological controls but are not suitable as excipients for human use. The second group included various oils that may give insight into improving TDF bioavailability but may be costly or difficult to formulate into a product suitable for human use. The third group included various compounds (EDTA, MP, PP, TPGS) that are GRAS U.S. Food and Drug Administration (FDA)–approved and suitable as excipients for human use.

### Effect of pH on TDF Stability *In Vitro*

**Supplementary Figure S1:**
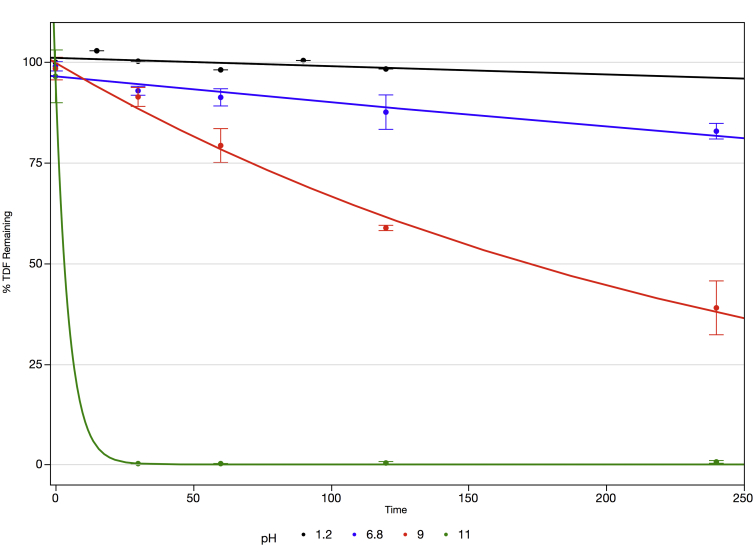
pH stability of TDF. About 1 mM TDF was incubated in triplicate at 37°C in SGF at pH 1.2 (black line) and in FaSSIF at pH 6.8 (blue line), 9 (red line), and 11 (green line). TDF was quantified by LC-MS/MS.

TDF has a pKa of 3.75 and a partition coefficient (log *P*) of 1.25. It is fairly water soluble (15.3 mg/mL at room temperature) and its solubility increases with decreasing pH.^31^ Based on these chemical properties and *in vitro* intestinal permeability, TDF is classified according to Biopharmaceutics Classification System as a class 3 substance, defined by high solubility and low permeability.^32^ Supplementary Figure S1 demonstrates the stability of TDF in buffer solutions of varying pHs. TDF is highly stable at pH 1.2, as may be encountered in the stomach, with a half-life in excess of 55 h. TDF is moderately stable at pH 6.8, as may be encountered in the intestine, with a half-life of 16.6 ± 3.4 h. The observed pH stability agrees with earlier reports of half-lives at 37°C of >150 h at pH 2.2 and 8 h at pH 7.4.^33^ TDF is not stable at higher pHs above the physiological range where the half-life of TDF is 2.9 ± 0.2 h at pH 9 and 3.4 ± 6.1 min at pH 11. Although the increasing instability of TDF at higher pHs has no biological significance, it does have relevance during subsequent formulation product development.

### Degradation of TDF by PL *In Vitro* and Effect of Inhibitors

**Figure 1:**
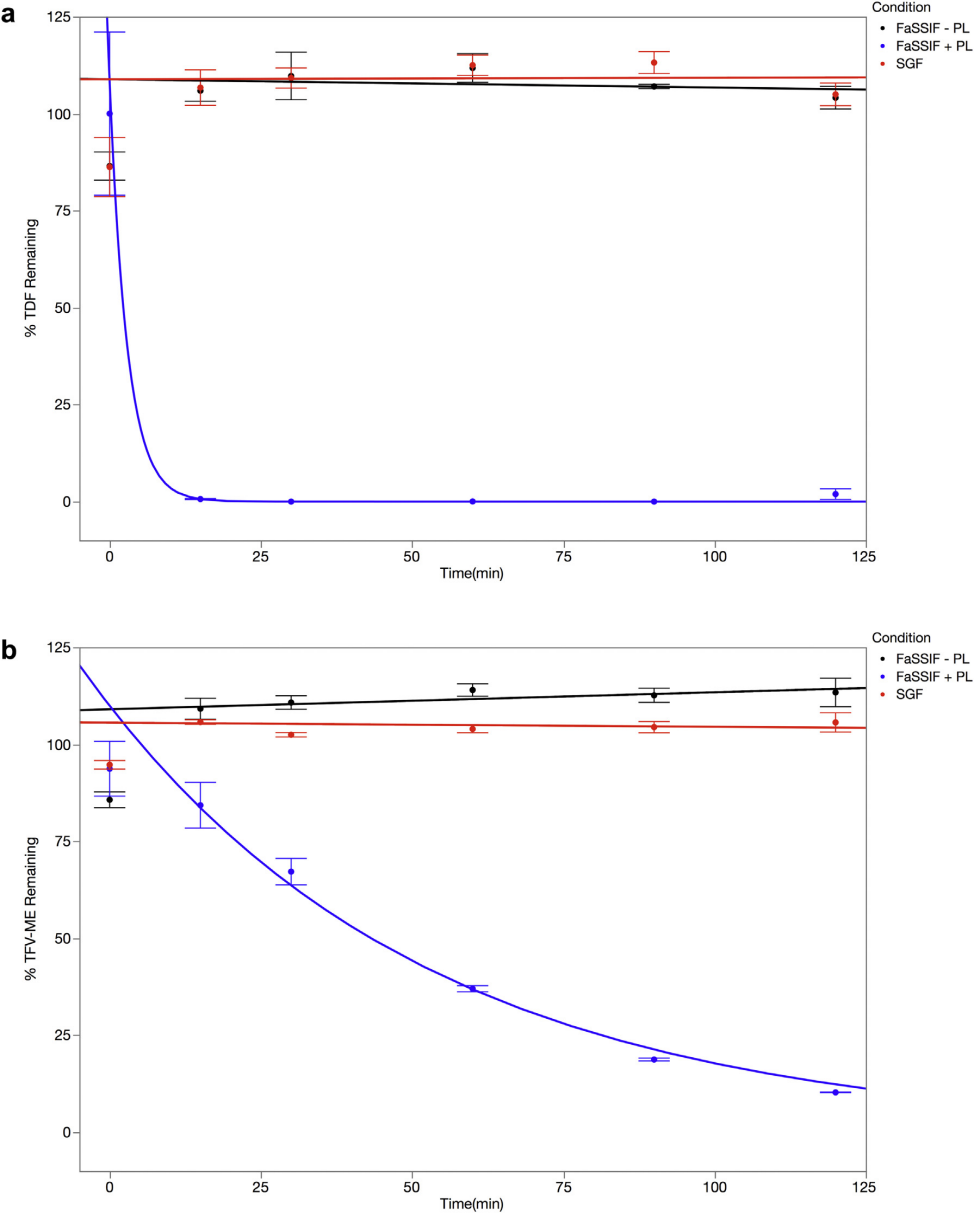
Hydrolysis of TDF and TFV-ME in SGF, FaSSIF, and FaSSIF with PL. About 10 μM TDF (a) or 10 μM TFV-ME (b) were incubated at 37°C in triplicate in SGF (red line), FaSSIF (black line), and FaSSIF with PL (blue line). TDF or TFV-ME was quantified by LC-MS/MS.

Bioavailability of TDF is influenced by fatty food intake,^10^, ^11^, ^12^ suggesting that fats may protect TDF by acting as alternative substrates for endogenous lipases and esterases. Therefore, we studied the effects of pancreatic lipase enzymes on the stability of TDF and TFV-ME in SGF and FaSSIF.^27^ TDF at 10 μM is stable in both SGF and FaSSIF (Fig. 1a), as would be anticipated from the pH stability results. Additionally, 1 mM TFV-ME is stable in SGF and FaSSIF (Fig. 1b), a previously unreported finding. PL is a semi-purified mixture of enzymes extracted from porcine pancreas to treat pancreatic insufficiency^34^ and was used here as a surrogate for human pancreatic activity. The addition of PL to FaSSIF (Fig. 1) resulted in rapid degradation of both TDF and TFV-ME with half-lives of 2.0 ± 2.3 and 38.0 ± 2.0 min, respectively, signifying that TDF and TFV-ME will be degraded quickly *in vivo* by pancreatic enzymes.

The pancreas secretes a host of digestive enzymes with broad specificity to degrade esters.^16^ PL is composed primarily of lipases, amylases, and serine proteases including chymotrypsin. Substrate specificities of lipases and CEs show significant overlap^35^, ^36^ and are thus both potentially capable of hydrolyzing the ester bonds in TDF. In addition, serine proteases have also been shown to have esterase activity^37^, ^38^ and may in turn hydrolyze TDF. Therefore, we tested the ability of a variety of substances and mixtures thereof to inhibit the degradation of TDF by PL in FaSSIF.

Van Gelder and Deferme^21^ had previously demonstrated that specific ester mixtures protect TDF against degradation in rat intestinal homogenates, most likely by inhibiting mucosal esterases. One of these ester mixtures, EM1, was composed of PP, octyl acetate, and ethyl caprylate in a 2:2:1 molar ratio. PP, a GRAS component of EM1, also protected TDF in rat intestinal homogenates when tested alone. We decided to test the ability of EM1 and PP to protect TDF in the presence of PL in FaSSIF. In addition to EM1 and PP, we tested MP, a PP congener, given that both PP and MP are CE inhibitors.^39^ A variety of oils were also tested, including soybean, safflower, sesame, olive, grape seed, corn, and coconut oil. We also evaluated the serine protease inhibitor PMSF and EDTA because of their ability to inhibit enzymatic activities of CEs, cholinesterases, and A-esterases.^40^

As shown in Table 1, 10% coconut oil and 10% soybean oil increased TDF stability by 2.79 ± 1.16-fold and 1.34 ± 0.54-fold, respectively. While 1 mM EDTA plus 10 mM EM1 and 10 mM EM1 alone increased TDF stability 1.83 ± 0.77-fold and 1.60 ± 0.63-fold, respectively, PP or MP together or alone did not protect TDF. The lack of PP activity implies that EM1 derives its stabilizing property from octyl acetate and ethyl caprylate.

### Degradation of TDF in Human and Rat Intestinal Extracts *In Vitro* and Effect of Inhibitors

**Figure 2:**
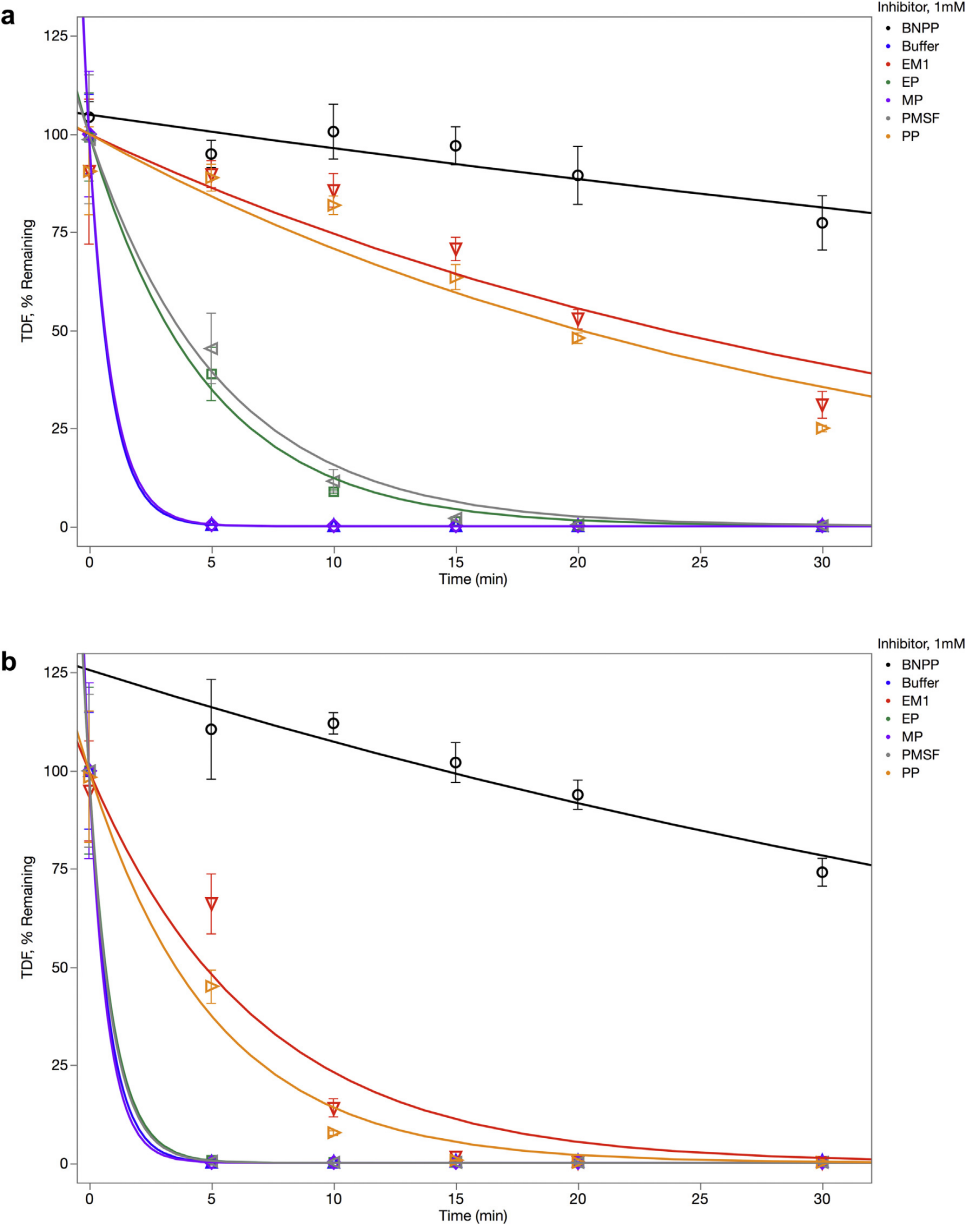
Stability of TDF in human and rat intestinal S9 preparations and effect of inhibitors. About 10 μM TDF was incubated at 37°C in triplicate with inhibitors using human S9 (a) and rat S9 (b). Inhibitor concentrations are the following: 10 μM BNPP, 100 μM PMSF, 0.05% wt/vol EM1, 1 mM PP, 1 mM MP, and 1 mM EP. TDF was quantified by LC-MS/MS. (Notes: The curves for buffer and MP overlap in the top panel. The curves for buffer, EP, MP, and PMSF overlap in the bottom panel.)

To examine the effects of luminal enzymes on TDF catabolism, TDF stability in rat and human intestinal S9 extracts was examined in the presence and absence of various inhibitors. As shown in Figure 2 (Supplementary Table S1), TDF is rapidly degraded in both human and rat intestinal extracts with half-lives of 0.62 ± 1.29 and 0.58 ± 1.79 min, respectively. BNPP, a CE inhibitor,^41^ was the most effective inhibitor tested and was found to increase the half-life of TDF to 81 ± 20 and 44 ± 7 min in human and rat S9, respectively. This finding indicates that CEs play a significant role in the hydrolysis of TDF within intestinal mucosa. Both EM1 and PP substantially protected TDF from hydrolysis. EM1 resulted in TDF half-life of 23.6 ± 2.3 and 4.7 ± 0.4 min in human and rat S9, respectively; PP resulted in TDF half-life of 20.1 ± 1.8 and 3.5 ± 0.4 min in human and rat S9, respectively. Notably, TDF in the human S9 was afforded a greater degree of protection by these inhibitors, with significantly longer half-life than in the rat S9, reflecting the species-related differences in the intestinal content of CEs. In agreement with this finding, earlier studies^42^, ^43^ demonstrated higher levels of CEs and other esterases in rat intestines relative to human intestines. PMSF, EP, and MP did not show any substantial inhibition of TDF hydrolysis in either S9 extracts (Fig. 2). These results indicate that of the FDA-approved excipients tested, PP has the highest potential to increase TDF stability and, in turn, TDF bioavailability by inhibiting CE hydrolysis of TDF.

### Effect of Efflux Inhibitors on TDF Transport *In Vitro*

The uptake and transport of a drug across the intestinal mucosa is governed by the drug’s intrinsic chemical properties, its bioconversion during transport, and efflux via P-gp.^17^, ^18^ Thus, we evaluated the bidirectional transport (apical to basolateral and vice versa) of TDF across monolayers of canine kidney MDR1-MDCK and human intestine Caco-2 cell lines. We set the *in vitro* study conditions to avoid transport saturation and to stay in the range of *K*_d_ of the P-gp receptor or *K*_m_ of the metabolizing enzymes of interest as well as to stay within the solubility of the compounds in the media used.

To clearly establish the role of efflux and its inhibition on the transport of TDF, we used the MDCK-MDR1 cell line, which overexpresses P-gp. Although these cells are of canine renal origin, they better delineate the role of P-gp in drug efflux than Caco-2 cells.^44^, ^45^ Table 2 shows that the basolateral (B) to apical (A) transport (B > A) of TDF across an MDCK monolayer is significantly higher than the A to B transport (A > B) with a *P*_app, B>A_ of 295.0 nm/s ± 6.1 and a *P*_app, A>B_ of 6.7 nm/s ± 0.1. The table also shows that the addition of 2 μM of GF918^23^ to the apical side substantially reduced the B to A transport (*P*_app, B>A_ = 34.1 ± 0.4), which suggests a key role for P-gp-mediated drug efflux in limiting the absorption of TDF across the gut. These data for TDF translate to an efflux ratio of 44.2 and 1.5 in the absence and presence of GF918. Furthermore, Table 2 shows that TFV-ME is only poorly transported with a *P*_app, B>A_ of only 8.8 nm/s ± 0.6 and an efflux ratio of 2.1. Finally, the table also shows that TFV-ME transport is also P-gp-mediated as 2 μM GF918 reduces *P*_app, B>A_ to 5.0 nm/s ± 0.6, which reduces the efflux ratio to 0.9. These results emphasize the importance of blocking conversion of TDF to TFV-ME and confirm that efflux inhibitors have the potential to increase the absorption and, in turn, bioavailability of TDF *in vivo* by decreasing efflux back into the intestinal lumen.

**Figure 3:**
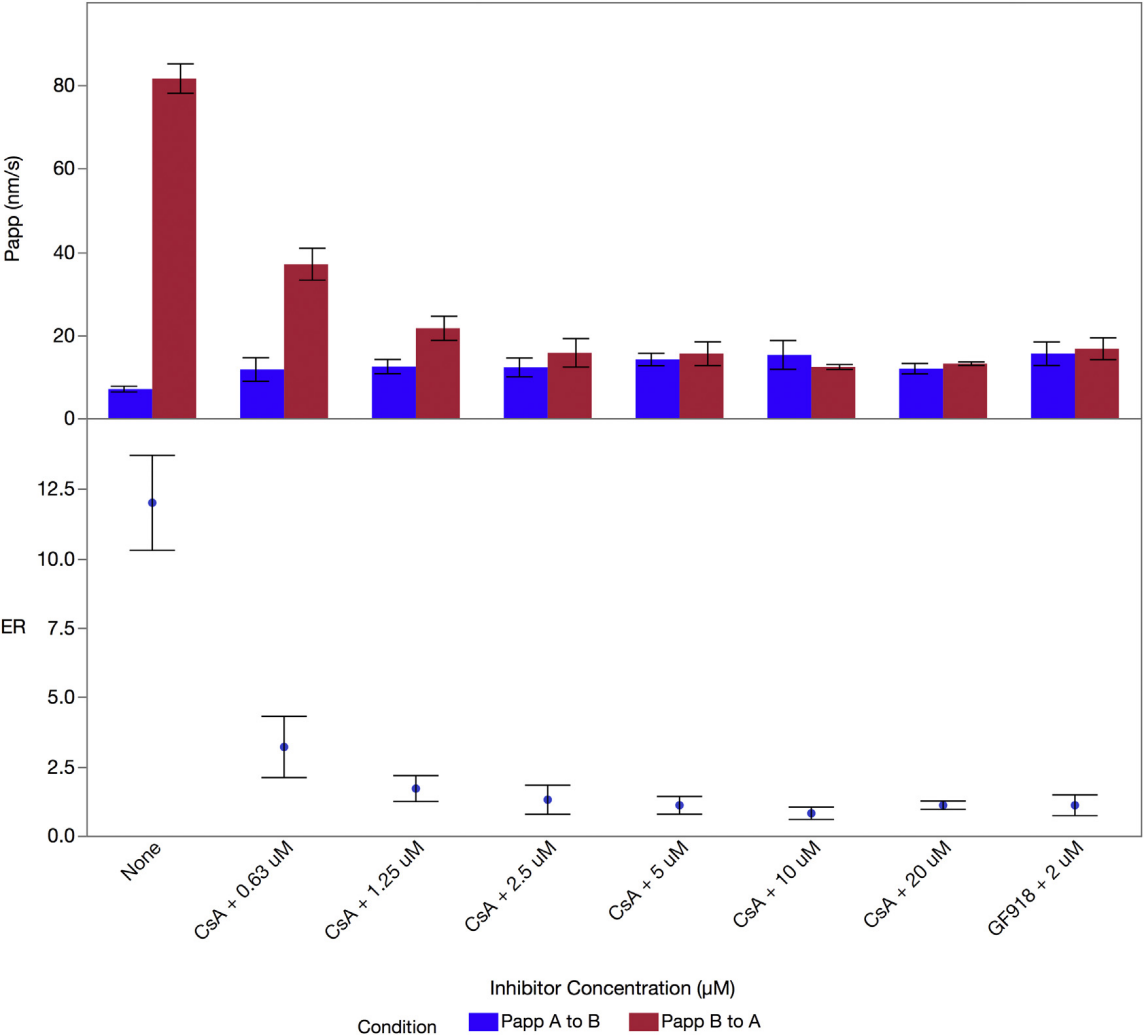
Transport of TDF across Caco-2 cell monolayers and effect of P-gp inhibitor CsA. About 10 μM TDF was placed on the apical (A) or basolateral (B) side of the monolayer, and transport of TDF across the monolayer was measured in triplicate from both compartments. CsA, at indicated concentrations, was added to the apical side. Samples were incubated for 120 min. TDF amounts were determined by LC-MS/MS. Top panel: A to B transport rate (blue bars) and B to A transport (red bars). Bottom panel: The data in the top panel transformed into efflux ratios. Error bars are ±1 SD.

We then used the Caco-2 cell line to explore intestinal transport in the presence of a broader complement of degradative enzymes, a scenario more representative of the small intestine.^46^, ^47^ Figure 3 (top panel) (Supplementary Table S2) shows the bidirectional transport rates of TDF across a Caco-2 cell monolayer as a function of the concentration of CsA, a P-gp inhibitor,^24^ as well as in the presence of 2 μM GF918. These studies also demonstrated that TDF efflux is regulated largely by the P-gp transporter in Caco-2 cells. Figure 3 (bottom panel) displays the data from the top panel as the efflux ratio, confirming that P-gp-mediated efflux plays a major role in the transport across the Caco-2 monolayer with an efflux ratio of 12.0 and 1.1 before and after addition of P-gp inhibitors, respectively. Therefore, efflux is likely to influence the intestinal absorption of TDF.

### Effect of Excipient Addition on the Stability and Transport of TDF Across Caco-2 Cells

**Figure 4:**
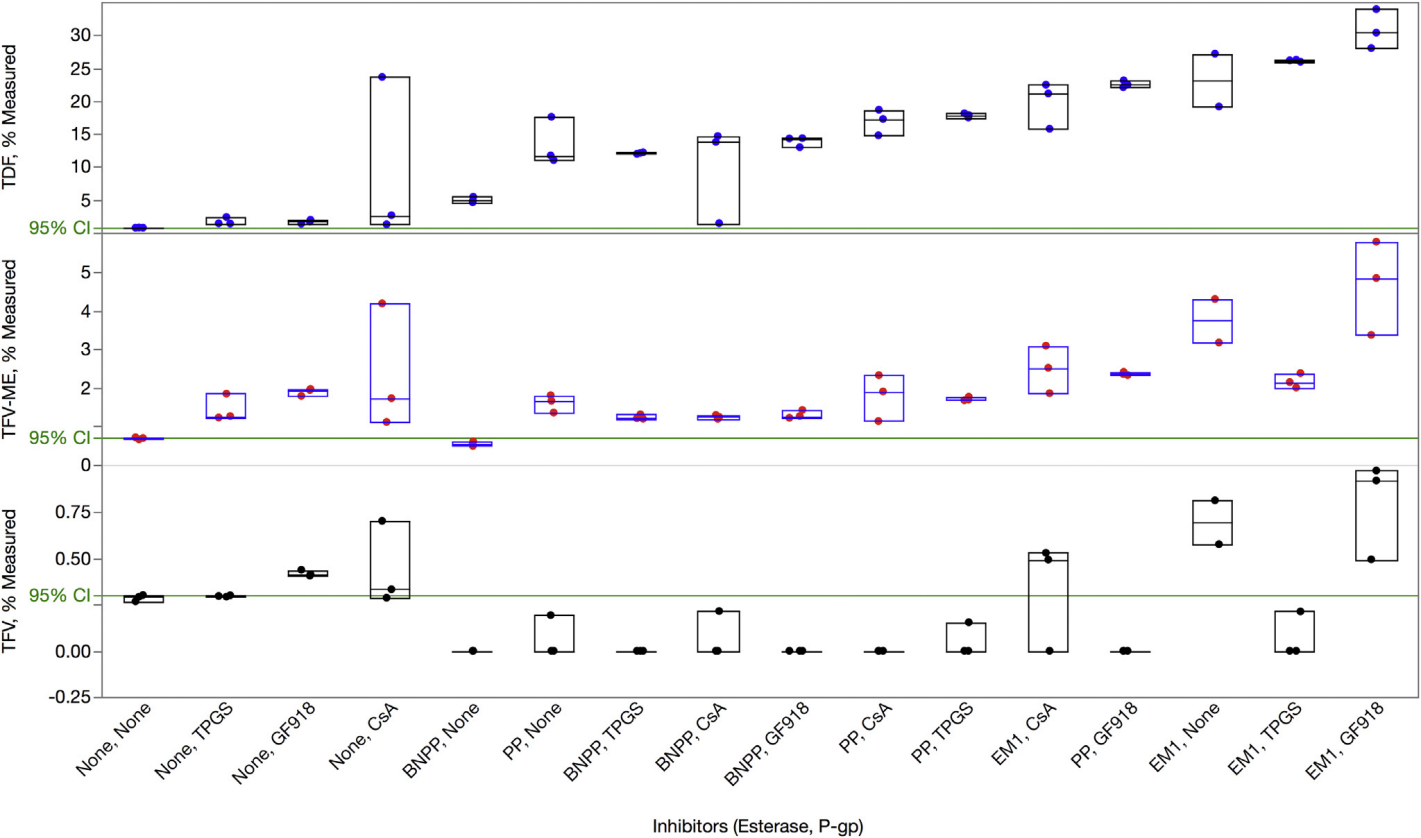
Influence of combinations of esterase and P-gp inhibitors on the apical to basolateral transport of TDF, TFV-ME, and TFV across Caco-2 monolayers. About 10 μM of TDF, TFV-ME, or TFV and inhibitors were added to the apical side in triplicate. Inhibitor concentrations are the following: 1 mM BNPP, 1 mM PP, 0.05% wt/vol EM1, 0.5 mg/mL TPGS, 10 μM CsA, and 2 μM GF918. Samples were incubated for 120 min. Amounts of all TFV species were determined by LC-MS/MS from both apical and basolateral chambers. The *y*-axis represents the amount of TDF, TFV-ME, and TFV measured on the apical side after 120 min. The data are plotted on the *x*-axis in ascending order based on median TDF values.

Having demonstrated the stabilizing effects of CE inhibitors and the involvement of efflux in TDF transport, we then tested combinations of these agents for their ability to enhance the transport of TDF and its metabolites (TFV-ME and TFV) across Caco-2 cell monolayers. Figure 4 shows the results from assessing EM1, PP, and BNPP as esterase inhibitors and CsA, GF918, and TPGS as efflux inhibitors.^48^, ^49^ These results are plotted in ascending order of efficacy with increasing TDF transport based on the median values of TDF measured in the basolateral compartment.

Table 3 presents the median values of basolateral measurements for TDF, TFV-ME, and TFV. The column labeled “TDF Fold Increase” shows the impact of esterase and efflux inhibitors on overall transport of TDF to the basolateral compartment. Esterase inhibitors alone enhanced transport of TDF by as much as 29.5-fold, whereas efflux inhibitors alone enhanced transport of TDF by at most 2.2-fold. If the addition of an esterase and an efflux inhibitor was purely additive, we would have expected TDF transport to increase to approximately 32-fold. However, we saw a synergistic effect from combining both types of inhibitors and TDF transport increased 38.7-fold when EM1 and GF918 were added. We also saw a 20.4-fold increase in transport of all tenofovir-containing species (TFV-family) in the presence of both types of inhibitors. This represents the potential for delivering TFV into systemic circulation. We showed that the following treatments were statistically different from the control based on 95% CI—EM1 + GF918, PP + TPGS, BP + TPGS, EP + TPGS, and TPGS—whereas the following treatments were not statistically different from the control based on 95% CI—CsA alone, TPGS alone, EM1 alone, and CsA + BNPP.

From the perspective of reformulating TDF with FDA-approved GRAS excipients, PP alone showed a 14.9-fold increase in transport of TDF and TPGS alone showed a 1.8-fold increase. The combination of PP and TPGS produced a transport enhancement of 22.8-fold for TDF and 11-fold for the TFV-family. This combination represents the best combination of FDA-approved GRAS excipients for increasing transport.

### Oral Bioavailability Enhancement Studies in Rat

**Figure 5:**
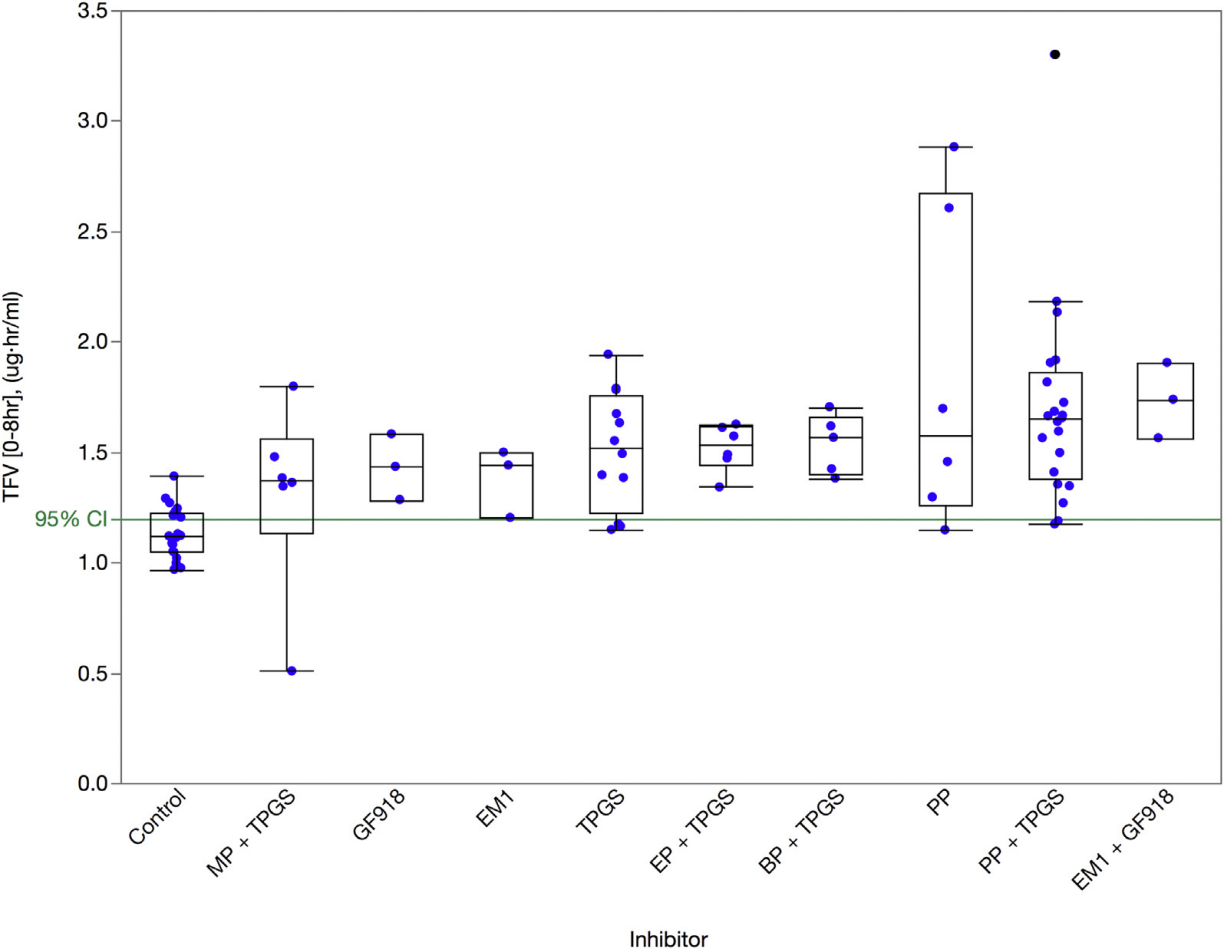
Rat PK following oral administration of TDF (25 mg/kg) with and without inhibitors using 6 animals per group. Inhibitor concentrations are the following: 40 mg/kg total for BP, EP, MP, and PP; 100 mg/kg TPGS; 8 mg/kg EM1; and 3 mg/kg GF918. Blood samples were taken at 20, 40, 60, 80, 100, and 120 min and then at 4 and 8 h. TFV was quantified by LC-MS/MS. The green horizontal line represents the 95% CI for the control.

Figure 5 (Supplementary Table S3) summarizes work on various inhibitors on the TFV PK profile and illustrates how the esterase inhibitors EM1, BP, EP, MP, and PP; the efflux inhibitors GF918 and TPGS; and their combinations impact TDF oral bioavailability (AUC_0-8h_) averaged across 6 fasted, male Sprague-Dawley rats. Although the rank order of enhancement was slightly different, the rat study recapitulated the *in vitro* Caco-2 data (Fig. 4 and Table 3). TPGS by itself increases bioavailability by 1.36-fold. Various combinations of TPGS with BP, EP, MP, and PP increased bioavailability between 1.22- and 1.47-fold, with PP having the largest impact. TDF in combination with EM1 and GF918 increases bioavailability by 1.55-fold. We showed that the following treatments were statistically different from the control based on 95% CI—EM1 + GF918, PP + TPGS, BP + TPGS, EP + TPGS, and TPGS alone—whereas the following treatments were not statistically different from the control based on 95% CI—PP alone, GF918 alone, EM1 alone, and MP + TPGS. This combination is not suitable for TDF reformulation as it contains non-GRAS excipients but gives an indication of the optimization potential using esterase and efflux inhibitors.

**Figure 6:**
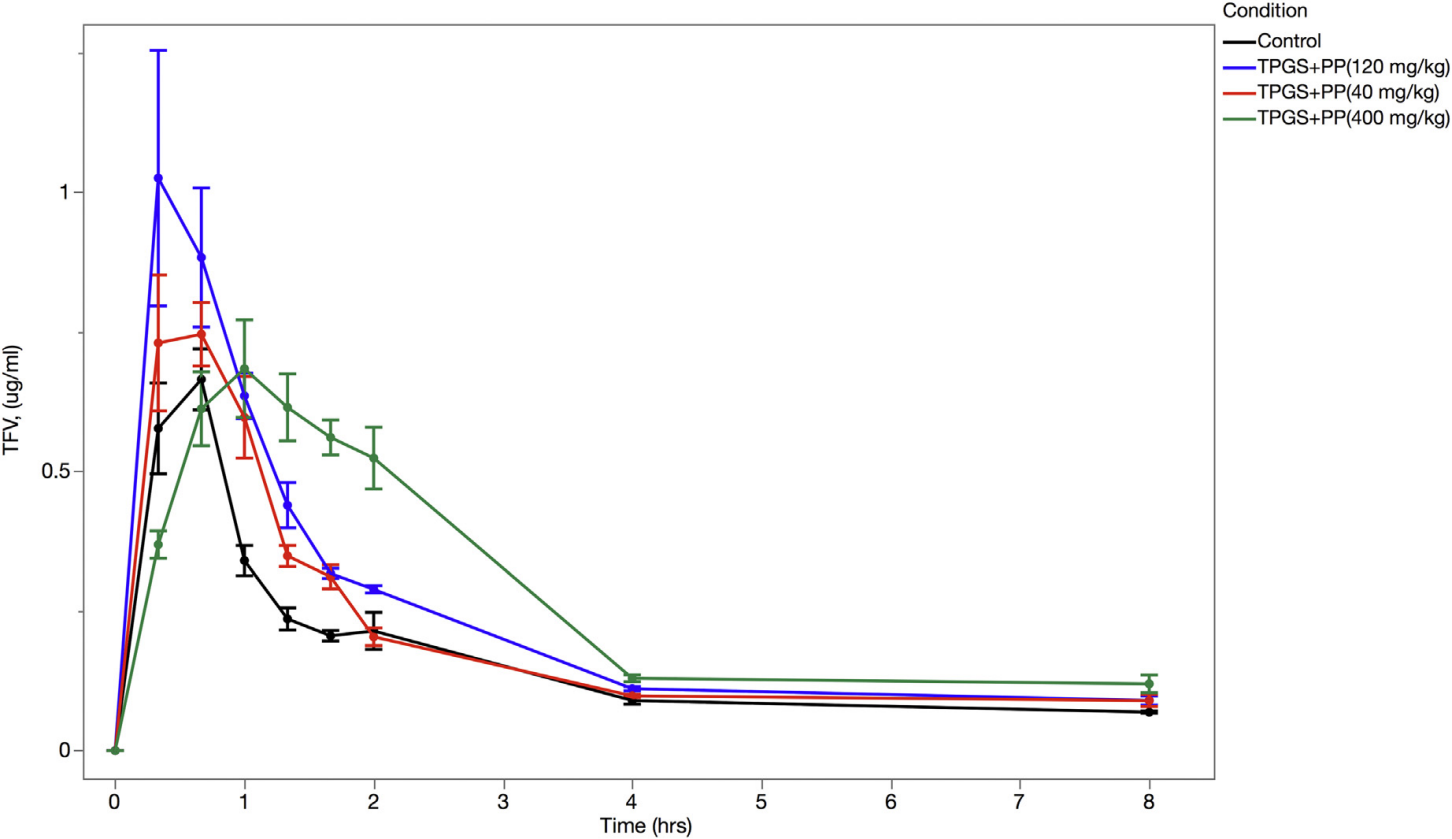
Rat PK following oral administration of TDF (25 mg/kg) with and without inhibitors. Full PK profile of TFV blood levels in the absence (black line) and presence of 40, 120, or 400 mg/kg PP and 100 mg/kg TPGS using 6 animals per group. Blood samples were taken at 20, 40, 60, 80, 100, and 120 min and then at 4 and 8 h. TFV was quantified by LC-MS/MS.

Figure 6 (Supplementary Table S4) explores the impact of PP dose ranging and shows the PK profile of TFV levels averaged across 6 fasted, male Sprague-Dawley rats against control. Each test set of animals was administered, by oral gavage, 25 mg/kg of TDF and 100 mg/kg TPGS. In addition, the animals were dosed with PP at 40, 120, or 400 mg/kg.

When TDF was administered alone, we observed a *T*_max_ of 0.56 ± 0.17 h, a *C*_max_ of 0.66 ± 0.13 μg/mL, and an AUC of 1.14 ± 0.02 μg·h/mL. The literature shows *T*_max_ values ranging from 0.25 to 0.4 h, and when these studies are normalized for dose, they show *C*_max_ and AUC values of 0.66 μg/mL and 1.49 μg·h/mL, respectively.^50^ These minor differences may arise from the age of the rats used, the exact protocol, and so on. PP shows a dose-dependent broadening of the PK profile and a fold increase on TFV AUC_[0-8h]_ of 1.65, 1.71, and 1.93 μg·h/mL for 40, 120, and 400 mg/kg PP, respectively. At 400 mg/kg PP, we also see a shift to a longer *T*_max_ of 1.00 ± 0.37. These changes are consistent with an increased and sustained availability of TDF through the inhibition of TDF hydrolysis by PP and the inhibition of efflux by TPGS.

Supplementary Tables S3 and S4 include a comparison of the AUC_0-8h_ for TFV given intravenously (IV) of 3.20 mg·h/mL, which represents 100% bioavailability. Therefore, in our rat studies, the dynamic range for bioavailability of TFV extends from 35% for the control group to 100% for the IV group. The PK measurements for PP and TPGS, our best GRAS combination, translate to 51.7%–60.4% bioavailability at 40 mg/kg and 400 mg/kg PP, respectively. The higher dose of PP affords a moderate advantage but poses a substantial challenge when developing a formulation for use in humans. A TDF:TPGS:PP dose in rats of 25 mg/kg TDF, 100 mg/kg TPGS, and 40 mg/kg PP translates to a dose ratio in humans of ∼1:1:0.8, given maximum allowed amounts and gut volume differences between rats and humans. This tablet would contain 300 mg of TDF, 300 mg of TPGS, and 240 mg of PP based on the current approved TDF dose of 300 mg.

## Discussion

TFV is a nucleotide analog of adenosine 5′-monophosphate that inhibits HIV reverse transcriptase.^11^, ^33^, ^51^ Because of TFV’s low oral bioavailability, it was developed and brought to market as the diester prodrug TDF, the active ingredient in VIREAD^®^.^8^, ^9^, ^33^

Orally administered TDF appears in the blood stream almost exclusively as TFV because TDF is hydrolyzed quickly in both the gut and blood stream by several hydrolases of the lipase and CE families. The oral bioavailability of TDF is 25% when administered under fasted conditions and 39% when administered after a high fat meal.^10^ However, this type of meal is not representative of the average diet in RLS. A Ugandan study that administered TDF with a more moderate fat meal that is typical of diets in RLS (i.e., 650 kcal and approximately 19 g of fat) found only marginally increased exposure to TFV compared to fasting.^52^ Kearney et al.^11^ as well as Gilead^53^ similarly noted that eating a light meal, which is more likely the case for a majority of patients, did not have a significant effect on TFV PK as compared to a fasted state. In agreement with these assertions, Geboers et al.^54^ did not detect a food effect on AUC from consuming a nutritional drink, although changes were observed in the blood PK profiles as well as in the intestinal lumen’s protection of TDF and TFV-ME as determined by direct sampling of the subject’s gut. To date, no published studies have been carried out that use active excipients to increase the bioavailability of TDF.

The goal of this research was to increase the oral bioavailability of TDF through the addition of excipients appropriate for use in human oral dosage forms. Known factors that affect a drug’s oral bioavailability are its solubility, metabolism, and absorption efflux back into the intestine. We have shown (unpublished data) that none of the excipients used in oral dosage form products from either innovator or generic^53^ affect TDF bioavailability. Because TDF is a highly soluble compound,^31^ we focused on identifying excipients that would inhibit metabolism and prevent intestinal efflux. We specifically studied agents included in the FDA’s GRAS list to eliminate additional regulatory hurdles.

TDF is very stable at pHs found in the stomach (ca. pH 2) and intestine (ca. pH 6.8),^19^, ^33^ with TDF half-lives being substantially greater than the rat or human gut transit times.^55^ Thus, chemical instability is not the cause of TDF’s modest bioavailability. Results shown in Supplementary Figure S1 demonstrate that TDF is hydrolyzed rapidly in simple buffers at pH values above the physiological range (≥pH 9) and, as such, will impact subsequent tablet formulation development.

After leaving the stomach, TDF is exposed to enzymes secreted from the pancreas via the common bile duct and from the small intestine itself. The pancreatic juice contains pancreatic lipase and CEs,^16^ which can hydrolyze TDF. In addition, the intestines express CE2.^56^, ^57^ Human intestinal fluid has been shown to contain a variety of proteins such as IgA heavy and light chain, alpha-amylase, carboxypeptidase A1, carboxypeptidase B, elastase IIA, elastase IIIA, anionic trypsin, phospholipase A2, triacylglycerol lipase, and lithostathine-1-alpha^40^; some of these enzymes and PDEs are believed to hydrolyze TDF to TFV-ME and then to TFV.^11^, ^54^ Unfortunately, the specific enzymes that hydrolyze TDF and TFV-ME have not been determined, but the hydrolases family, which includes lipases and CEs, is assumed to play a key role.^35^, ^54^ The situation is further confounded by the broad substrate specificity of lipases and CEs^36^, ^58^, ^59^, ^60^ and the fact that even some serine proteases such as trypsin and chymotrypsin have esterase activities.^37^, ^38^ As such, the task of protecting TDF from hydrolysis by a specific enzyme will be difficult.

Several empirical studies have supported the general feasibility of our approach toward protecting TDF against degradation. Van Gelder and Deferme^21^ showed that the addition of a variety of esters and parabens inhibits TDF hydrolysis in intestinal extracts. Notably, one such mixture, EM1, composed of the lipids octyl acetate and ethyl caprylate as well as the ester PP, protected TDF from intestinal esterase hydrolysis.

As shown in Figures 1a and 1b, we observed rapid degradation of TDF and TFV-ME in the presence of porcine PL, a crude pancreatic extract that primarily contains lipase, amylase, and chymotrypsinogen. TFV-ME is more stable than TDF to degradation by PL, a result consistent with that reported by Geboers et al.^54^ As shown in Table 1, coconut oil, soybean oil, EM1, and EM1 combined with the chelator EDTA extended the half-life of TDF, whereas the other oils (safflower, sesame, or olive), MP, PP, EDTA alone, or the serine protease inhibitor PMSF did not.

While the results with EM1 were expected based on previous findings,^21^ the influence of coconut oil was surprising. Coconut oil, unlike most other oils tested, contains triglycerides and mainly lauric and myristic acids with smaller proportions of capric, caproic, caprylic, oleic, palmitic, and stearic acids.^61^ EM1 contains octyl acetate and ethyl caprylate, which have similar formulas (C_10_H_20_O_2_); thus, medium chain fatty acids may stabilize TDF to the esterase hydrolysis. Coconut oil as well as caprylic, lauric, and myristic esters are all GRAS listed, but add substantial complexity to tablet formulation.

BNPP, EM1, and PP emerged as the most potent inhibitors of TDF hydrolysis in human and rat intestinal S9 extracts as demonstrated in Figure 2 (top and bottom panels). Within the MP, EP, and PP ester series, PP was the best inhibitor under our conditions (Supplementary Table S1). Given that the half-life for PP was similar to EM1 in both human and rat S9 extracts, octyl acetate and ethyl caprylate do not appear to play a substantial role in protecting TDF from enzymatic hydrolysis. These results agree with the findings of Jewell et al.^39^ that showed MP and EP to be preferred substrates for hCE1, which is expressed in the liver, and PP and BP to be preferred substrates for hCE2, which is expressed predominantly in the intestine. Although a large number of potent CE inhibitors have been described in the literature,^41^ none of these appear on FDA’s GRAS list. The studies described here suggest that the FDA-approved GRAS product, PP, is the best and most appropriate excipient to include in TDF formulation.

Drugs administered orally are absorbed by the intestinal enterocytes where many are then actively effluxed back to the intestinal lumen by efflux transporters. Efflux is primarily mediated by the adenosine triphosphate–binding cassette family of transporters, of which P-gp is one member.^17^, ^18^ Several chemicals, polymers, and detergents that inhibit efflux transport have been described here and in the literature,^18^ and many of these are included in FDA’s GRAS list. Table 2 shows that both TDF and TFV-ME—to a lesser extent—were substrates for P-gp efflux in a MDCK-MDR1 assay by showing that B to A transport of TDF was strongly blocked by the P-gp-specific inhibitor GF918 with the *P*_app, B>A_ dropping from 295.1 to 34.1 nm/s and from 8.8 to 5.0 nm/s for TDF and TFV-ME, respectively. We used this method because MDCK-MDR1 cell lines overexpress the P-gp transporter while expressing relatively low concentrations of esterases. Furthermore, we were able to confirm that TDF is a substrate for P-gp-mediated efflux in a Caco-2 cell assay.^21^ Despite the data and the discussion around the pros and cons of using potent and specific P-gp inhibitors to affect TDF absorption, no research to date has empirically tested the feasibility of enhancing oral absorption of TDF with P-gp inhibitors.

In our Caco-2 assays (Fig. 3 and Supplementary Table S2), TDF showed a 7.0 nm/s *P*_app, A>B_ and an 81.6 nm/s *P*_app, B>A_. This large baseline efflux ratio of 12 was reduced to an efflux ratio of 1.3 and 1.1 with the addition of 2.5 μM CsA^24^ or 2 μM GF918,^23^ respectively, which is consistent with a P-gp transporter-mediated process. Our A to B *P*_app_ value is identical to that reported by Tong et al.^26^ Furthermore, the magnitude of the efflux ratio we observed is comparable to that determined by Tong et al.^26^ when taking into account the acknowledged variability in these types of experiments.

Although we demonstrated (Fig. 4 and Table 3) that GF918 and CsA were the best efflux inhibitors (2.2- and 1.9-fold increase over control, respectively) and showed utility in helping us define the potential dynamic range of inhibition, they are not appropriate for use in a TDF dosage formulation. TPGS^48^, ^49^ was shown to be the best GRAS-listed P-gp inhibitor and increased TDF transport by 1.8-fold. Similarly, although we were able to demonstrate that EM1 was the best esterase inhibitor and showed utility in helping us define the potential dynamic range of inhibition with a 29.5-fold increase over control, it is not appropriate for use in a TDF dosage formulation. PP however was shown to be the best GRAS-listed esterase inhibitor and increased TDF transport by 14.9-fold. When both types of these GRAS-listed inhibitors were included, TDF transport increased by 22.8-fold.

Based on the aforementioned *in vitro* results, we examined the effects of various combinations of esterase and P-gp inhibitors on TDF oral bioavailability in fasted, male Sprague-Dawley rats. As noted above, in the Results section on the oral bioavailability in rats, a TDF:TPGS:PP dose in rats of 25 mg/kg TDF, 100 mg/kg TPGS, and 40 mg/kg PP translates to a dose ratio in humans of ∼1:1:0.8 given maximum allowed amounts and gut volume differences between rats and humans. Based on human equivalent dose corrected for body surface area using a scaling factor of 6.2 (FDA guidance ref, Table 1), a 300 mg human dose with a 70 kg person scales to approximately 26 mg/kg in rat.^62^ Figure 5 (Supplementary Table S3) showed similar results to those found in Caco-2 cell studies. EM1 in combination with GF918 increased AUC over control by 1.55-fold. PP among all the parabens (BP, EP, and MP) performed the best and increased bioavailability in the presence of TPGS by 1.47-fold. Figure 6 and Supplementary Table S4 demonstrate a bioavailability dose response to PP, with 400 mg/kg PP and TPGS increasing bioavailability by 1.72-fold over controls. Qualitatively, we saw that an increase in AUC with the addition of esterase inhibitors broadened the PK curves.

As we noted in the Results section, the *in vitro* and *in vivo* experiments had inherently different dynamic ranges. Specifically, the bioavailability study is theoretically limited to a 3-fold improvement. We can show that by normalizing the dynamic range of the 2 types of experiments, the results from these experiments are complementary. In the Caco-2 study, PP and TPGS yield a 52% normalized maximal response by setting the minimum value to the control and the maximum value to the results with EM1 + GF918. In the rat study, PP and TPGS, at the highest concentrations tested, yield a 39% normalized maximal response by setting the minimum value to the control and the maximum value to the IV results.

As referenced earlier in this article, resource-limited markets are looking in earnest for ways to maintain or improve quality and reduce the cost of treatment regimens. Activities such as dosage reformulation or dose reduction offer these benefits. However, in order to actually move the market toward such measures, incentives must be included in the price to satisfy both buyers and suppliers. For buyers to take on the logistics of replacing one product with another, a substantial cost reduction would be required from launch onwards to generate interest in switching to new reformulated products. For suppliers to be incentivized, they would need to see a short-term premium embedded into the cost of manufacturing to justify the effort of developing and registering the new product. Based on our market analyses, we set the reformulation success criteria at a 25% reduction in API per dose or 225 mg once daily (CHAI Market Analysis, internal). From an economic perspective, this could dramatically impact cost savings per year and has the potential to serve millions of patients. Furthermore, demonstrating a positive outcome from a clinical proof of concept would stimulate research on other products and translate into more cost savings in the future.

## Conclusion

The ester prodrug TDF, which is widely used today for first-line ART in RLS, has a low oral bioavailability of approximately 25%. Improving TDF’s bioavailability can result in significant cost savings and greater affordability in the global effort to combat HIV/AIDS. Two significant factors that limit oral bioavailability are the degradation of TDF in the gastrointestinal tract and the P-gp-mediated efflux of the absorbed drug back into the intestinal tract. In these *in vitro* and *in vivo* studies, we investigated pharmacological agents that inhibit enzymatic degradation of TDF and its efflux. We have identified an esterase inhibitor, PP, and an efflux inhibitor, TPGS, that may enhance TDF bioavailability. Both PP and TPGS are FDA-approved GRAS excipients. This will enable us to advance to human clinical trials without complicating the design of the human studies. Using a rat model, we have demonstrated that TDF formulations with PP and TPGS increase oral bioavailability by 1.72-fold. Given that esterase activity in the rat intestine exceeds that in the human intestine, we expect to see even better relative bioavailability in humans. Validating this work in humans represents an opportunity through reformulation to reduce dose while maintaining the efficacy of the innovator product. The authors recognize that TPGS does not have a strong record for improving bioavailability on its own. However, given the cumulative *in vitro* and *in vivo* finding for TDF when combined with TPGS and PP, this study suggests that further studies in humans are warranted.

Since our group has extensive experience costing commodity drugs for RLS, we are able to calculate with reasonable certainty how much of a product’s price is due to raw materials, API manufacturing, formulation, overhead, and profit. Given the size of the market for products containing TDF, we determined that a reduction in the standard dose of TDF from 300 to 225 mg through a 33% improvement in bioavailability would lead to $50-75 million in annual savings (CHAI Market Analysis, internal). These cost savings would significantly extend the treatment of HIV/AIDS to larger populations in RLS and help to combat the global AIDS epidemic.

## Figures and Tables

**Table 1:** Hydrolysis of TDF by PL and Effect of Enzyme Inhibitors

Condition	*t*_1/2_ (min)	*t*_1/2_ Error	Fold Increase	Fold Error
Control, FaSSIF	6.35	0.90	1.00	0.28
10% (vol/vol) coconut oil	17.70	4.82	2.79	1.16
10% (vol/vol) soybean oil	8.52	2.19	1.34	0.54
10% (vol/vol) safflower oil	7.11	1.63	1.12	0.42
10% (vol/vol) sesame oil	4.81	1.06	0.76	0.27
10% (vol/vol) olive oil	4.49	0.84	0.71	0.23
Control, transport media	7.59	0.76	1.00	0.20
1 mM EDTA, 10 mM EM1	13.92	4.49	1.83	0.77
10 mM EM1	12.11	3.57	1.60	0.63
1 mM EDTA	8.06	2.52	1.06	0.44
50 μM EDTA, 2 μM PMSF	7.41	1.03	0.98	0.23
10 mM PP	6.79	1.06	0.89	0.23
50 μM EDTA	6.46	0.98	0.85	0.21
5 mM PP, 5 mM MP	5.83	0.96	0.77	0.20
10 mM MP	5.15	0.79	0.68	0.17

About 10 μM TDF was incubated in triplicate at 37°C in FaSSIF containing PL with and without inhibitors. TDF was quantified by LC-MS/MS.

**Table 2:** Transport of TDF and TFV-ME Across MDCK Cell Monolayers and Effect of P-gp Inhibition

Direction	0 μM GF918		2 μM GF918	
	TDF, *P*_app_ (nm/s)	TFV-ME, *P*_app_ (nm/s)	TDF, *P*_app_ (nm/s)	TFV-ME, *P*_app_ (nm/s)
A to B	6.7 (0.14)	4.1 (0.20)	22.5 (0.57)	5.3 (0.61)
B to A	295.0 (6.1)	8.8 (0.60)	34.1 (0.42)	5.0 (0.58)
Efflux ratio	44.2	2.1	1.5	0.9

About 5 mM TDF or 5 mM TFV-ME with or without 2 μM P-gp inhibitor GF918 was added in triplicate to the apical side of monolayers. Samples were incubated for 120 min. TDF and TFV-ME were quantified by LC-MS/MS from both apical and basolateral compartments. Numbers in parentheses are the SEM.

**Table 3:** Influence of Combinations of Esterase and P-gp Inhibitors on the Apical to Basolateral Transport of TDF, TFV-ME, and TFV Across Caco-2 Monolayers

Esterase Inhibitor	P-gp Inhibitor	% Measured Basolateral					Fold Increase	
		TDF	TDF, Lower 95% Mean	TDF, Upper 95% Mean	TNF-ME	TNF	TDF	TFV Family
None	None	0.78	0.75	0.84	0.70	0.29	1.0	1.0
	TPGS^a^	1.45	0.37	3.14	1.27	0.30	1.8	1.7
	CsA^a^	1.47	−21.91	40.32	1.25	0.29	1.9	1.7
	GF918	1.72	0.99	2.40	1.95	0.41	2.2	2.3
BNPP	None	4.88	3.92	6.06	0.55	0.00	6.2	3.1
	TPGS	12.12	11.81	12.39	1.21	0.00	15.4	7.5
	CsA^a^	14.69	−8.37	28.33	1.19	0.00	18.7	8.9
	GF918	14.31	11.95	15.82	1.27	0.00	18.2	8.8
PP	None	11.73	4.49	22.41	1.66	0.00	14.9	7.5
	TPGS	17.87	17.01	18.64	1.69	0.00	22.8	11.0
	CsA	17.27	12.03	21.79	1.91	0.00	22.0	10.8
	GF918	22.49	21.28	23.87	2.36	0.00	28.7	14.0
EM1	None^a^	23.17	−27.77	74.11	3.75	0.70	29.5	15.5
	TPGS	26.17	11.00	28.60	2.15	0.00	33.3	15.9
	CsA	22.47	25.74	26.52	3.10	0.53	28.6	14.7
	GF918	30.37	23.40	38.15	4.86	0.92	38.7	20.4

About 10 μM of TDF, TFV-ME, or TFV and inhibitors were added to the apical side in triplicate. Inhibitor concentrations are the following: 1 mM BNPP, 1 mM PP, 0.05% wt/vol EM1, 0.5 mg/mL TPGS, 10 μM CsA, and 2 μM GF918. Samples were incubated for 120 min. Amounts of all TFV species were determined by LC-MS/MS from both apical and basolateral chambers and reported as the median.

a Treatments that were not statistically different from control.
