# Supplementary material for: Development of a Novel Formulation That Improves Preclinical Bioavailability of Tenofovir Disoproxil Fumarate
Source: J Pharm Sci. 2017 Mar;106(3):906–19. doi: 10.1016/j.xphs.2016.12.003 (PMC5320394; doi:10.1016/j.xphs.2016.12.003)
Supplement: Supplementary Tables S1-S4 [file mmc1.docx]

Table S1

| **S9 Source** | **Group** | **Rate**  **Constant,**  **minutes^-1^** | **Std. Error** | **t_1/2_,**  **minutes** | **t_1/2_, Error** |
| --- | --- | --- | --- | --- | --- |
| Human | Buffer | -1.123 | 2.351 | 0.62 | 1.29 |
| Human | BNPP | -0.009 | 0.002 | 81.20 | 19.71 |
| Human | EM1 | -0.029 | 0.003 | 23.56 | 2.32 |
| Human | PP | -0.035 | 0.003 | 20.06 | 1.82 |
| Human | PMSF | -0.185 | 0.017 | 3.74 | 0.34 |
| Human | EP | -0.210 | 0.020 | 3.30 | 0.32 |
| Human | MP | -1.065 | 1.763 | 0.65 | 1.08 |
|  |  |  |  |  |  |
| Rat | Buffer | -1.200 | 3.712 | 0.58 | 1.79 |
| Rat | BNPP | -0.016 | 0.003 | 43.99 | 7.21 |
| Rat | EM1 | -0.146 | 0.013 | 4.74 | 0.42 |
| Rat | PP | -0.196 | 0.019 | 3.54 | 0.35 |
| Rat | EP | -1.023 | 1.529 | 0.68 | 1.01 |
| Rat | PMSF | -1.064 | 1.876 | 0.65 | 1.15 |
| Rat | MP | -1.297 | 6.007 | 0.53 | 2.48 |

Table S2

| **Inhibitor** | **Inhibitor Conc.**  **(µM)** | **Direction** | | | | **E.R.** | **ER**  **Error** |
| --- | --- | --- | --- | --- | --- | --- | --- |
|  |  | **A to B** | | **B to A** | |  |  |
|  |  | **Papp**  **(nm/s)** | **Papp**  **SD** | **Papp**  **(nm/s)** | **Papp**  **Error** |  |  |
| CsA | 0.00 | 6.98 | 0.69 | 81.60 | 3.53 | 12.0 | 1.71 |
| CsA | 0.63 | 11.70 | 2.83 | 37.00 | 3.83 | 3.2 | 1.11 |
| CsA | 1.25 | 12.40 | 1.72 | 21.60 | 2.92 | 1.7 | 0.47 |
| CsA | 2.50 | 12.20 | 2.25 | 15.70 | 3.41 | 1.3 | 0.52 |
| CsA | 5.00 | 14.10 | 1.50 | 15.50 | 2.86 | 1.1 | 0.32 |
| CsA | 10.00 | 15.20 | 3.44 | 12.30 | 0.58 | 0.8 | 0.22 |
| CsA | 20.00 | 11.90 | 1.27 | 13.10 | 0.40 | 1.1 | 0.15 |
| GF918 | 2.00 | 15.50 | 2.83 | 16.70 | 2.63 | 1.1 | 0.37 |

Table S3

| **Condition** | **TFV AUC (μg.hr/ml)** | | | | | | | **Fraction**  **of IV** |
| --- | --- | --- | --- | --- | --- | --- | --- | --- |
|  | **Mean** | **SD** | **SEM** | **Lower**  **95%**  **Mean** | **Upper**  **95%**  **Mean** | **Median** | **Median**  **Fold**  **Increase** |  |
| Control | 1.14 | 0.11 | 0.02 | 1.09 | 1.19 | 1.12 | 1.00 | 35.0% |
| EM1 0.1M + GF918 | 1.74 | 0.17 | 0.10 | 1.31 | 2.16 | 1.74 | 1.55 | 54.3% |
| PP 0.1M + TPGS | 1.70 | 0.46 | 0.10 | 1.49 | 1.91 | 1.65 | 1.47 | 51.7% |
| PP 0.1M^*^ | 1.85 | 0.72 | 0.30 | 1.09 | 2.61 | 1.58 | 1.40 | 49.2% |
| BP 0.1M + TPGS | 1.54 | 0.13 | 0.06 | 1.37 | 1.71 | 1.57 | 1.40 | 48.9% |
| EP 0.1M + TPGS | 1.52 | 0.11 | 0.04 | 1.41 | 1.63 | 1.53 | 1.36 | 47.8% |
| TPGS | 1.51 | 0.27 | 0.08 | 1.34 | 1.68 | 1.52 | 1.36 | 47.5% |
| EM1^*^ | 1.38 | 0.16 | 0.09 | 0.99 | 1.77 | 1.44 | 1.28 | 45.0% |
| GF918^*^ | 1.43 | 0.15 | 0.09 | 1.06 | 1.80 | 1.43 | 1.28 | 44.8% |
| MP 0.1M + TPGS^*^ | 1.31 | 0.43 | 0.17 | 0.86 | 1.76 | 1.37 | 1.22 | 42.9% |
|  |  |  |  |  |  |  |  |  |
| IV TNF | 3.21 | 0.16 | 0.06 | 3.04 | 3.37 | 3.20 | 2.85 | 100.0% |

Table S4

| **Condition** | **TFV AUC**  **(μg∙hr/ml), Median** | **Fold Increase** | **Fraction of IV** |
| --- | --- | --- | --- |
| PP (400 mg/kg) + TPGS | 1.93 | 1.72 | 60.4% |
| PP (120 mg/kg) +TPGS | 1.71 | 1.53 | 53.5% |
| PP (40 mg/kg)+ TPGS | 1.65 | 1.47 | 51.7% |
